# Association Between Dynapenia, Central Obesity, and Physical Function in Young Adults: A Cross-Sectional Study

**DOI:** 10.7759/cureus.65285

**Published:** 2024-07-24

**Authors:** Aruna Raju, Niveatha S, Jean Fredrick, Madhavan Chandran

**Affiliations:** 1 Physiology, All India Institute of Medical Sciences, Kalyani, Kalyani, IND; 2 Physiology, Mahatma Gandhi Medical College and Research Institute, Puducherry, IND

**Keywords:** young adults, muscle strength, physical function, obesity, dynapenia

## Abstract

Background: Muscle strength is recognized as a key indicator of overall health and can help identify the risk of cardiometabolic disease. This study explores the relationship between low muscle strength, central obesity, and physical function among young adults.

Methods: We conducted a cross-sectional study from a convenience sample of 513 adults aged 18-25. Participants' anthropometric measures such as height, weight, and circumferences of both waist and hip were measured, and body mass index (BMI) was calculated. Subjects were tested for hand grip strength (HGS) using hand dynamometry. Relative hand grip strength (RHGS) was derived by dividing maximum HGS by BMI. Physical function was assessed using a six-minute walk test. The International Physical Activity Questionnaire (IPAQ) was administered.

Results: Overweight and obesity were present in 313 (61%) of the study population. Central obesity was observed in 194 (37.8%) of the population. RHGS showed a positive association with physical function and physical activity, and a negative association was observed with BMI and waist circumference. Low RHGS was categorized as < 25th percentile by gender. The non-dynapenic non-central obese group had higher physical function (644± 124.2) than others. There was no difference in the dynapenic and central obese groups. The dynapenic central obese group had significantly lesser physical function (424.9±69.1) than all other groups in both genders.

Conclusion: Our study supports the importance of early investigation of dynapenia, which can increase the risk of chronic disease and accelerate the development of physical limitations. Understanding how dynapenia and central obesity relate to low physical function is of growing importance in young adults, and it can play an important role in overall health.

## Introduction

Muscular strength is a powerful determinant of overall health. There is an inverse association between muscular strength, obesity, and cardiometabolic disease risk factors in young adults. Reduction in muscular strength is a well-recognized risk factor for poor physical function and morbidity, even in young adults. Sarcopenia is defined as a loss of muscle mass; however, muscle weakness can also occur due to a decrease in the intrinsic force-generating properties of the skeletal muscle. Therefore, Clark and Manini proposed the term "dynapenia as "poverty of strength," which refers to decreased muscle strength and power due to aging [1]. Nevertheless, the effects of decreased muscle strength and low muscle function are not limited to the elderly alone; even young adults are also vulnerable to the inevitable consequences of reduced strength, physical function, and fitness, so the concept of dynapenia should also be extended to include the younger population [2]. Dynapenia in young adults and children describes an acquired and treatable condition characterized by low muscular strength and physical functional limitations not caused by neurologic or muscular disease [3]. Most adolescents and young adults are physically inactive, and global trends in muscular fitness indicate that present-day young adults have less strength and are slower than previous generations [4]. The 'poverty of strength' is common in many children, young adults, and older populations. Physical inactivity and sedentary behavior are the causes of reduced muscle strength and power seen in young adults. Reduction in muscle strength at any age predisposes individuals to functional limitations, poor physical performance, activity-related injuries, and other detrimental outcomes. Young adulthood is a critical period for lifestyle change, like an increase in unhealthy weight and a decrease in physical activity, which affects overall health [5]. Young adults with low muscular strength are less likely to participate in physical activity and more likely to have disease risk factors and poor cardiometabolic health.

In addition to muscle mass, other factors may contribute to the components of muscle strength and play a role in the decrease in muscle function. Obesity and low physical fitness are more commonly associated with functional disability [6]. Studies have shown that muscle of obese persons have greater muscle mass and strength than nonobese persons. However, muscle quality, a measure of muscle strength relative to muscle mass, is lower in obese persons than in nonobese persons [7]. Being overweight and obese, even at a young age, is a definite deterrent to physical activity. Waist circumference (WC) is often used as the surrogate measure of central or visceral adiposity, affecting muscle strength. It is reported that muscle architecture is deranged due to the infiltration of intramuscular fat, impairing muscle quality and, thus, leading to lower physical performance, strength, and function [8]. Studies have demonstrated that dynapenic central obesity has an increased risk of metabolic disorders, falls, physical disability, cardiovascular dysfunction, and mortality when compared to either dynapenia or central obesity alone [9]. The combined effect of dynapenia and central obesity can predispose to poor physical function and are associated with structural and functional changes in skeletal muscle. Understanding the link between obesity and the risk of reduced muscle strength as a proxy for future dynapenia is essential.

Moreover, previous research regarding dynapenic obesity investigates this condition in the elderly population. Thus, this study aims to identify the relationship between hand grip strength (HGS), central obesity, and physical function in young adults. This is important in this context as it is susceptible to intervention.

## Materials and methods

This cross-sectional study involved a convenient sample of 513 healthy young adults (263 males and 250 females) aged 18-25. Participants were selected using random sampling methods. All subjects provided written informed consent before participating in the study. The study excluded individuals with wrist, hand, or leg injuries, as well as those with hypertension or diabetes mellitus.

The study was approved by the Institutional Human Ethics Committee MGMCRI, Puducherry, vide letter no (MGMCRI/IRC/04/2020/32/IHEC/179) on 25/9/2020. Written informed consent was obtained for participation in the study and use of the patient data for research and educational purposes.

Anthropometry

The study participants' body mass index (BMI) was calculated from their height and weight. WC was measured according to the International Society for the Advancement of Kinanthropometry (ISAK) recommendation.

The new Asian BMI criteria were used to classify obesity. Normal weight was between 18.5 and 22.99 kg/m^2^ BMI. Individuals with a BMI from 23 to 24.99 kg/m^2^ were classified as overweight. A BMI of or exceeding 25 kg/m^2^ was classified as obesity. Additionally, abdominal obesity was defined using WC measurements. For men, a WC of 90 cm or greater indicated abdominal obesity. For women, the cut-off was set at 80 cm or above.

A short version of the self-reported International Physical Activity Questionnaire (IPAQ) was employed to evaluate physical activity levels. This tool measures how much time individuals spent engaging in physical activities over the previous week. It specifically focuses on the frequency and duration of walking and moderate and vigorous physical exercise periods lasting at least 10 minutes each. Using standardized scoring criteria, we classified participants into three groups based on their activity levels: "low" (indicating physical inactivity), "moderate," and "high." The total metabolic equivalent (MET) scores were computed by aggregating the MET values for walking, moderate-intensity, and vigorous-intensity activities [10].

Physical function was assessed using a six-minute walk test, a submaximal exercise used to assess aerobic capacity and endurance. Two cones were placed 30 meters away, and the distance covered by the participants in 6 minutes was measured in meters. Two recordings were taken. The subjects were encouraged verbally to perform maximum, and the highest distance covered in two trials was used for analysis [11].

Assessment of maximum grip strength on subjects' non-dominant hands was done using calibrated hand dynamometers. The devices were tested with known weights to ensure accuracy. The width of the dynamometer was adjusted for each participant's comfort. During testing, subjects stood upright, holding the dynamometer with their arm extended slightly away from their body. They performed three separate trials, with a minimum 30-second rest between attempts. All subjects were provided with verbal encouragement to promote maximum effort. The highest value from the three attempts was recorded for analysis. To account for body size differences, relative grip strength was determined by dividing the maximum grip force by the subject's BMI [12].

Statistical analysis

Statistical analysis was done using IBM SPSS Statistics for Windows, Version 21 (Released 2012; IBM Corp., Armonk, New York, United States). The normality of the data was checked using the Shapiro-Wilk test. Continuous data were represented as mean and standard deviation. Pearson correlation coefficient was used to find the association between relative hand grip strength (RHGS) and BMI, WC, and total METs. One-way ANOVA with Bonferroni correction was done to find the difference in RHGS between the BMI categories. An independent t-test was done to find the difference between the normal and obese groups categorized by WC. p<0.05 was considered statistically significant. Low RHGS was categorized as < 25th percentile by gender and was considered dynapenic. Central obesity was defined by Asian cut-off points of WC, and based on this, it is categorized into four groups: non-dynapenic non-central obese group, dynapenic group, central obese group, and dynapenic central obese group. A general linear model with Bonferroni correction was done to find the difference in physical function between these groups.

## Results

A total of 513 participants (263 males and 250 females) were included in the study. The mean age of the participants shows no difference; though males were significantly taller and heavier than females, there was no significant difference in BMI between genders. WC was also similar between males and females. Significant gender differences were observed across all physical function measures. Males demonstrated higher absolute HGS and RHGS compared to females. In the six-minute walk test, males covered significantly more distance than females. Similarly, total METs were substantially higher in males than in females, as represented in Table 1.

**Table 1:** Demographic characteristics and physical performance measures based on gender. Data represented as mean±standard deviation; METs: metabolic equivalents

Parameters	Total	Male (263)	Female (250)	p-value
Age	23.43±1.1	23.41±1.08	23.41±1.13	0.673
Height (cm)	163.6±8.7	167.4± 8.2	159.7±7.4	<0.001
Weight (kg)	70.1±14.7	70.1±14.7	65.23±16	<0.001
Body mass index (kg/m^2^)	25.25±5.35	24.99±4.7	25.53±5.8	0.261
Waist circumference (cm)	81.41±5.34	82.21±11.6	80.5±12.3	0.124
Hand grip strength (kg)	28.28±5.5	32.32±3.8	24.04±3.5	<0.001
Relative hand grip strength (kg/kg m^2^)	1.15±0.29	1.3±0.23	0.9±.24	<0.001
Six-minute walk test (m)	563±144.7	650.2±126.7	472.6±99.6	<0.001
Total METs	975.5±472.4	1190.5±481.21	749.8±339.6	<0.001

The frequency distribution of the study population based on Asian criteria of BMI category and WC is shown in Table 2, indicating that 313 (61%) of the total population were classified as overweight and obese. Central obesity, as indicated by WC, showed a marked gender difference. Of the study population, 194 (37.8%) exhibited central obesity. However, the prevalence was nearly twice as high in females 126 (50.4%), compared to males 68 (25.9%). This suggests a significantly higher risk of abdominal obesity among women in the study population.

**Table 2:** Frequency distribution based on BMI category and waist circumference.

Parameters	Total	Male (263)	Female (250)
Underweight	48 (9.4%)	25 (9.5%)	23 (9.2%) (23)
Normal weight	152 (29.6%)	73 (27.8%)	79 (31.6%)
Overweight	73 (14.2 %)	36 (13.7%)	37 (14.8%)
Obese	240 (46.8%)	129 (49%)	111 (44.4%)
Normal waist circumference	319 (62.2%)	195 (74.1%)	124 (49.6%)
Central obesity	194 (37.8 %)	68 (25.9 %)	126 (50.4%)

Table 3 demonstrates significant associations between RHGS and various anthropometric and physical function parameters (p < 0.001). Negative associations were observed between RHGS, BMI and WC. Conversely, there was a positive association between the six-minute walk test and total physical activity measured in METs. These relationships were consistent across both male and female participants.

**Table 3:** Association of relative hand grip strength with body mass index, waist circumference, and physical activity. p< 0.001; METs: metabolic equivalents

Relative hand grip strength	Male	Female
Body mass Index (kg/m^2^)	r = -0.775	r = -0.741
Waist circumference (cm)	r = -0.599	r = -0.642
Six-minute walk test (m)	r = 0.866	r = 0.838
Physical activity (Total METs)	r = 0.757	r = 0.812

**Figure 1:**
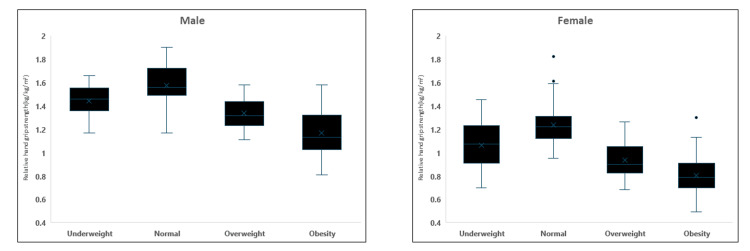
Relative hand grip strength based on the BMI category in males and females.

Figure 1 illustrates that RHGS was calculated and analyzed based on the BMI category in males and females. In both genders, RHGS values were lowest in the obese BMI category (1.16±0.17 in males, 0.80±0.13 in females). Statistically significant differences were observed between the normal BMI category and all other categories (p<0.001) for both males and females. Notably, in males, there was no significant difference in RHGS between the underweight (1.44±0.14) and overweight categories (1.33 ± 0.12), whereas, in females, a significant difference was noted between all four groups after Bonferroni correction.

**Figure 2:**
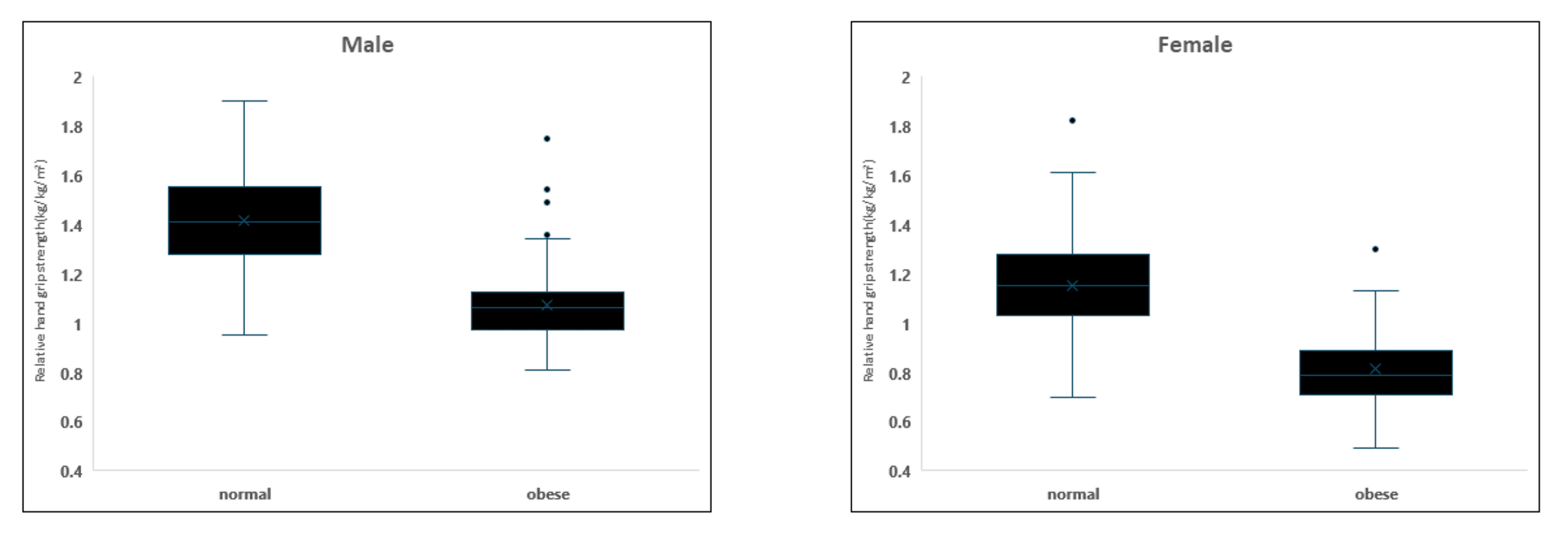
Relative hand grip strength based on the WC category in males and females. WC: Waist circumference

Figure 2 shows that RHGS was analyzed based on the WC category in males and females. Similar to BMI findings, RHGS values were lowest in the obese WC category for both genders. Statistically significant differences were found between the normal and obese WC categories (p<0.001) in both males and females. These results suggested that higher overall and central adiposity levels, as indicated by BMI and WC, respectively, were associated with lower RHGS in both males and females.

Dynapenia was defined as RHGS below the 25th percentile for each gender. The cut-off values in males were RHGS < 1.1337 and in females were RHGS < 0.7856. Central obesity was defined by WC of ≥ 90 cm in males and ≥80 cm in females. Physical function scores varied significantly among four groups: The non-dynapenic, non-central obese group demonstrated higher physical function (644 ± 124.2) compared to all other groups. No significant difference was observed between the dynapenic-only (528.8 ± 80.5) and central obese-only (496.5 ± 82.7) groups. Notably, individuals who were both dynapenic and centrally obese exhibited significantly lower physical function scores (424.9 ± 69.1) compared to all other groups, regardless of gender.

## Discussion

This study examined the relationships among muscle strength, central obesity, and physical function in young adults. Our findings supported our hypothesis: participants exhibiting both central obesity and low RHGS demonstrated reduced physical function. Males had higher absolute HGS, RHGS, physical function, and physical activity than females.

Low muscle strength and muscle mass in young people are mainly due to low accumulation of skeletal muscle mass primarily caused by physical inactivity and sedentary behavior rather than a reduction in muscle mass as occurs in aging [13]. Our results confirm previous reports that physical activity is a major determinant of muscle strength and quality [14]. Physical inactivity, particularly among adolescents and young adults, is a significant public health issue because it is a key modifiable risk factor for future chronic diseases [15]. Research has demonstrated clear links between insufficient physical activity, central obesity, and poor physical fitness with overall disease burden worldwide. These factors are also associated with impaired insulin sensitivity and reduced oxidative capacity in skeletal muscle. Addressing low levels of physical activity in younger populations is therefore crucial for preventing the development of chronic health conditions later in life. An increase in physical activity plays a crucial role in enhancing muscular strength and increasing muscle mass and also has a primary role in managing obesity. Interestingly, even without significant changes in body composition, an increase in physical activities enhances physical function capabilities in both individuals with normal weight and those who are obese. This suggests that improvements in their muscle strength and overall physical performance can be achieved through an increase in physical activity [16].

Research indicates that HGS may not accurately represent overall body strength in obese individuals, as obesity affects upper and lower body strength differently [17]. The association between absolute HGS and body weight suggests that using uncorrected HGS measurements to assess muscle strength could yield inconsistent results. Consequently, RHGS, which accounts for body weight, may better evaluate muscle strength in this population [18].

Central obesity measured by WC is a better predictor than BMI in identifying the risk of accelerated loss in muscle strength [19]. Adiposity can impact muscle ability even at young ages, independent of elevated muscle mass. Infiltration of fat in the muscle is the major determinant of muscle weakness. However, an increase in central obesity also influences muscle quality through insulin resistance and an increase in pro-inflammatory cytokines, such as tumor necrosis factor-alpha and interleukin-6, which are associated with lower muscle strength and physical function [6,20]. Our results show that physical function is decreased even in young adults with low HGS and central obesity. The concepts of dynapenic obesity, co-occurrence of dynapenia, and central obesity are to be discussed even in young adults because they can predict physical functional decline much earlier and other health outcomes. Dynapenic obesity is a double burden since the low level of muscle strength and increased fat together can increase the risk of cardiometabolic dysfunction even during early adulthood [21]. The association of increased fat infiltration with poor physical function has been reported in other studies. These findings suggest that an increase in muscle strength relative to per unit change in muscle mass, independent of fat accumulation, indicates improved muscle quality [22]. This observation carries significant clinical implications, as the functional limitations associated with central obesity are linked to poorer health outcomes, increased risk of chronic conditions, and reduced mobility. The resulting decrease in physical activity and energy expenditure may lead to further weight gain, perpetuating the cycle of obesity and negatively affecting skeletal muscle function. This interplay between obesity, reduced mobility, and declining muscle performance underscores the importance of maintaining muscle quality for overall health and functional capacity [23].

To our knowledge, no previous studies have investigated, especially, the effect of dynapenic central obesity on physical function in young adults. Identifying dynapenia in young adults is important because it is not only related to poor health outcomes and cardiac and metabolic risk, but it also indicates the start of functional limitations in neuromuscular activities and increased risk of physical activity-related injury, which increases the tendency toward sedentary behavior [24]. Thus, routine screening for RHGS and WC may be clinically valuable. In addition, improving strength through exercise among young adults would, in turn, facilitate improvements in physical fitness necessary for breaking the epidemic of obesity and improving health-related outcomes.

In line with our study, prior research has demonstrated that younger individuals with obesity tend to have a slower gait speed than normal-weight individuals. Additionally, findings of Vakula et al. revealed that obese young adults have reduced quadriceps muscle function and alterations in their gait. These changes can potentially hinder their ability to perform physical activity [25].

Only a few studies have investigated dynapenia in adolescents and children [26,27]. Our study supports the importance of early assessment of dynapenia, which can increase the risk of comorbidities and contribute to the early onset of physical disability. The concept of dynapenia, though associated with older populations, is becoming increasingly relevant for younger age groups. Temporal trends on muscular fitness among young adults reveal reduced muscular strength and overall fitness levels. This shift suggests that dynapenia should be considered a potential issue across a broader age spectrum rather than limited to elderly individuals.

Therefore, to prevent spontaneous tracking into late adulthood, obesity prevention through increased physical activity should be encouraged in young adults.

Limitations

Given this study's cross-sectional nature, it is impossible to establish causal relationships. Body composition measurements were not taken, which is the precise indicator of adiposity; WC and BMI in young adults serve as adequate measures for assessing adiposity. We have used self-reported questionnaires to evaluate physical activity levels rather than objective measurement devices such as accelerometers. This may have introduced potential bias due to participants' subjective interpretations. More precise instruments could have enhanced the data's validity and provided a more accurate representation of actual physical activity levels.

## Conclusions

This study highlights the significant relationship between low RHGS, central obesity, and reduced physical function in young adults aged 18-25. Our findings demonstrate that individuals with both low RHGS (dynapenia) and central obesity exhibit markedly lower physical function compared to their non-dynapenic, non-centrally obese counterparts across genders. These findings have important public health implications, highlighting the need for targeted interventions to improve muscular strength and reduce central obesity in young adults, potentially mitigating the risk of future chronic diseases and physical limitations.
